# Agreement Between Cone Beam Computed Tomography, Bone Sounding, Pulse–Echo Ultrasound, and Caliper Measurements for Gingival Thickness Assessment: A Method-Comparison Study

**DOI:** 10.3390/dj14090551

**Published:** 2026-09-02

**Authors:** Selma Dervisbegovic, Reinhard Gruber, Andrea Gamper, Stefan Nemec, Xiaohui Rausch-Fan, Gabriella Dvorak

**Affiliations:** 1 Clinical Division of Periodontology, University Clinic of Dentistry, Medical University of Vienna, 1090 Vienna, Austria; selma.dervisbegovic@meduniwien.ac.at; 2 Competence Center Oral Biology, University Clinic of Dentistry, Medical University of Vienna, 1090 Vienna, Austria; reinhard.gruber@meduniwien.ac.at; 3 Clinical Division of Pediatric Dentistry, University Clinic of Dentistry, Medical University of Vienna, 1090 Vienna, Austria; an_fa@gmx.at; 4 Clinical Division of Radiology, University Clinic of Dentistry, Medical University of Vienna, 1090 Vienna, Austria; stefan.nemec@meduniwien.ac.at; 5 Center for Clinical Research, University Clinic of Dentistry, Medical University of Vienna, 1090 Vienna, Austria; xiaohui.rausch-fan@meduniwien.ac.at

**Keywords:** gingival thickness, gingival phenotype, cone beam computed tomography, bone sounding, pulse–echo ultrasound, caliper measurement

## Abstract

**Background/Objectives**: Accurate assessment of gingival thickness (GT) is important for periodontal and implant treatment planning. **Methods**: In this method-comparison study based on prospectively collected clinical data from 2012, GT was assessed at standardized buccal sites using cone beam computed tomography (CBCT), bone sounding (BS), an earlier-generation pulse–echo ultrasound (US) device, and direct digital caliper (CA) measurement. Of 53 acquired CBCT scans, 12 (23%) were non-evaluable because the buccal soft tissues could not be reliably delineated, leaving 41 sites from 41 patients for analysis. A ±0.30 mm clinical comparison margin, based on the difference used in the original sample-size calculation, was applied to interpret agreement. **Results:** Mean comparator-minus-CBCT biases were −0.19 mm for BS (95% limits of agreement [LoA], −0.98 to 0.61 mm), −0.16 mm for US (95% LoA, −0.83 to 0.51 mm), and −0.71 mm for CA (95% LoA, −1.99 to 0.56 mm). Although BS and the tested US device showed smaller average differences from CBCT than CA, the LoA for all comparisons exceeded this margin. Phenotype classification was exploratory because only four sites were classified as thin by CBCT. **Conclusions**: BS and the tested pulse–echo US device showed smaller average differences from CBCT than CA; however, the limits of agreement for all comparisons exceeded this margin, so none of the comparator methods can be considered interchangeable with CBCT. The 23% non-evaluable CBCT rate and the device- and protocol-specific nature of the findings limit their generalizability to current-generation systems.

## 1. Introduction

Gingival thickness (GT) is a critical determinant of periodontal health, peri-implant stability, and esthetic outcomes. Adequate soft-tissue thickness supports predictable healing, protects underlying bone, and reduces the risk of recession or mucosal complications. In implant dentistry and periodontal surgery, GT influences flap management, connective tissue grafting decisions, and the long-term maintenance of soft tissues [1,2,3,4,5]. Understanding gingival phenotype and thickness allows clinicians to anticipate treatment outcomes, optimize surgical planning, and tailor interventions to individual patients, emphasizing the importance of accurate measurement in both clinical and research settings [6,7,8,9,10,11].

Techniques to assess GT include transgingival approaches such as bone sounding (BS) and transgingival probing [12], as well as ultrasound (US) and direct caliper (CA) measurement [13,14,15,16]; the invasiveness of caliper measurement depends on how the tissue is accessed, ranging from measurement on visible gingiva to measurement following surgical flap elevation. Direct-contact and surgical-exposure methods provide direct measurements but may cause patient discomfort and are subject to operator variability. Non-invasive imaging approaches such as ultrasound avoid tissue trauma but are prone to compression artifacts, operator dependence, and limited reliability in thin or mobile tissues. These inherent limitations highlight the need for measurement techniques that are both accurate and reproducible, offering practical applicability for routine clinical use [17,18,19,20,21,22,23].

Cone beam computed tomography (CBCT) has been investigated for soft-tissue evaluation because it can depict hard and soft tissues within the same dataset [24,25,26,27]. Recent studies have reported reproducible GT measurements under standardized acquisition conditions [28,29]. However, previous investigations often focused on palatal mucosa, individual anatomical regions, or comparisons with a single clinical method [25,30,31,32,33]. Consequently, evidence concerning agreement among several techniques applied to the same standardized buccal site remains limited. Agreement and association must also be distinguished: methods may correlate strongly while showing clinically important bias or wide limits of agreement.

A recent systematic review by Barausse et al. synthesized 17 clinical studies involving 371 patients and described applications of ultrasound imaging for preoperative planning and postoperative monitoring in implant dentistry [34]. The review emphasized substantial heterogeneity in transducer frequency, imaging mode, acquisition settings, probe positioning, and operator training. Findings obtained with single-point pulse–echo instruments, such as the Krupp SDM device used in the present study, should therefore not be generalized to contemporary high-frequency B-mode systems, color and power Doppler imaging, quantitative ultrasound, or strain elastography. These contemporary technologies have already been evaluated in human clinical studies, although routine integration remains limited by equipment availability, operator dependency, methodological heterogeneity, and the absence of standardized acquisition protocols.

Tavelli, Barootchi, and colleagues have used power Doppler ultrasonography to evaluate tissue perfusion and vascular healing at implant and palatal donor sites, to distinguish peri-implant health from disease through perfusion patterns, and to characterize soft-tissue elasticity and phenotype [35,36,37,38]. These studies demonstrate diagnostic and functional capabilities that differ fundamentally from the single-point A-mode pulse–echo measurement evaluated here.

The methodological novelty of the present study is the use of a customized thermoplastic splint with an incorporated radiopaque marker to co-localize the measurement site across CBCT, BS, pulse–echo US, and CA within the same clinical cohort. This design enabled a direct four-method comparison at a standardized buccal location, unlike recent studies that compared CBCT with one clinical method or evaluated different anatomical locations.

The primary objective was to quantify agreement between CBCT and BS, pulse–echo US, and CA for site-level GT measurements. Secondary objectives were to describe inter-examiner reliability, associations and distributional differences among methods, and exploratory phenotype classification. Because no independent reference standard such as histology was available, the study was treated as a method-comparison study rather than a diagnostic accuracy study, and no method was considered a true gold standard.

## 2. Materials and Methods

### 2.1. Study Participants

This report is a retrospective analysis of prospectively collected clinical data. Forty-one patients (31 women and 10 men; mean age, 42 ± 15 years; range, 23–74 years) were enrolled at the Division of Oral Surgery, University Clinic of Dentistry, Medical University of Vienna. Study preparation, including recruitment planning, began in December 2011. Following ethics approval on 27 January 2012, participant recruitment, informed consent, study-specific examinations, and data collection were conducted through July 2012. Consecutive patients requiring surgical treatment, including root resection or implant therapy, were examined. Exclusion criteria were pregnancy, lactation, and age below 18 years. The prospectively written protocol was approved by the Medical University of Vienna Ethics Committee (EK Nr. 1132/2011), and all participants provided written informed consent. Public study registration was completed retrospectively in September 2025. The study itself had been conducted on the basis of a prospectively written study protocol and ethics approval. The retrospective registration reflects the historical nature of the dataset and the later decision to complete and report the comparative analysis.

**Figure 1:**
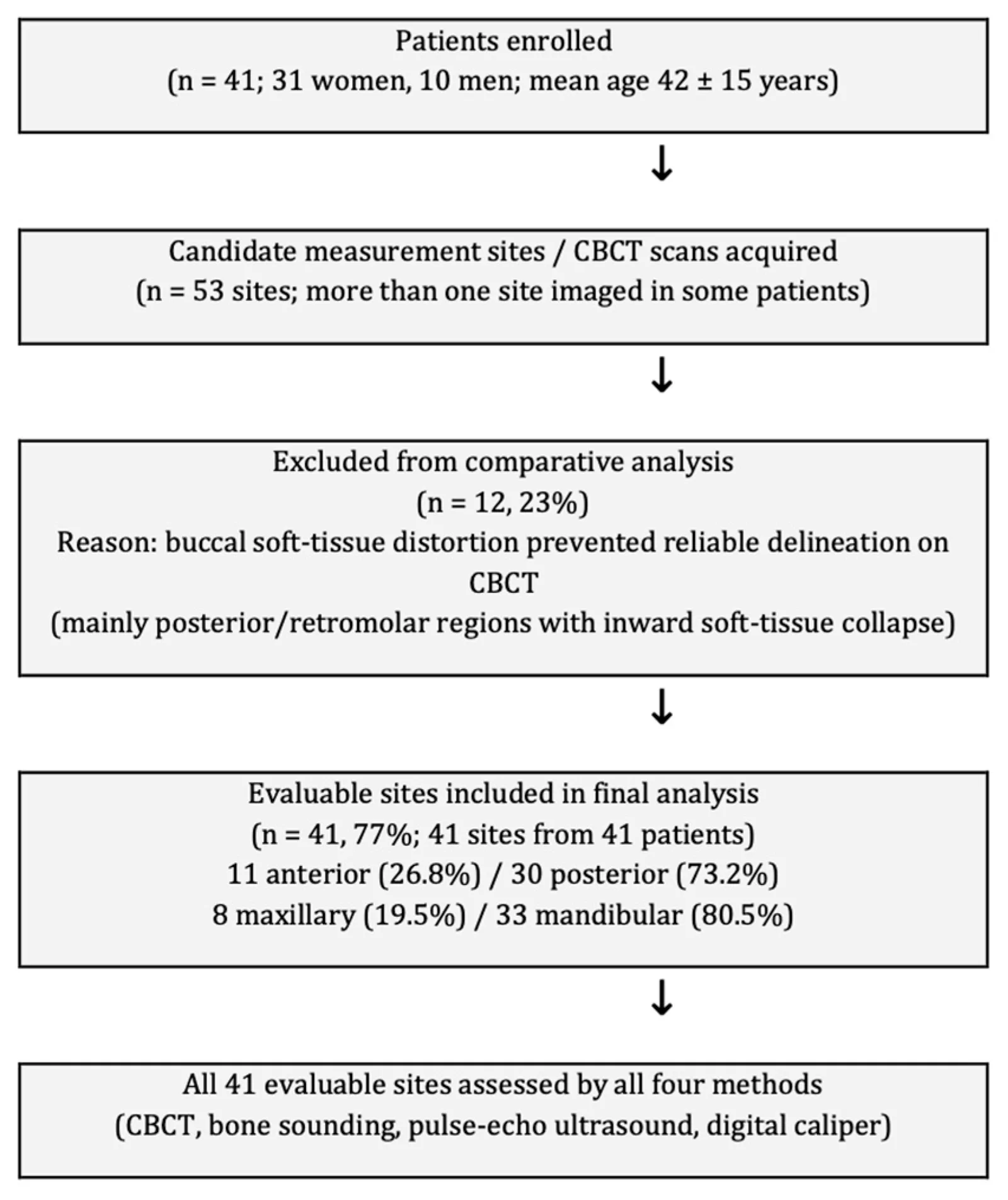
Participant and site flow diagram showing the relationship between enrolled patients, candidate measurement sites/CBCT scans, excluded scans, and the final evaluable dataset.

The unit of analysis was the measurement site. The 41 enrolled patients contributed 53 candidate sites and corresponding CBCT scans because more than one site was imaged in some patients. Twelve scans (23%) were excluded before comparative analysis because buccal soft-tissue distortion prevented evaluable measurements. The final analysis therefore comprised 41 evaluable sites from 41 patients (Figure 1).

### 2.2. Clinical and Radiological Assessments

**Figure 2:**
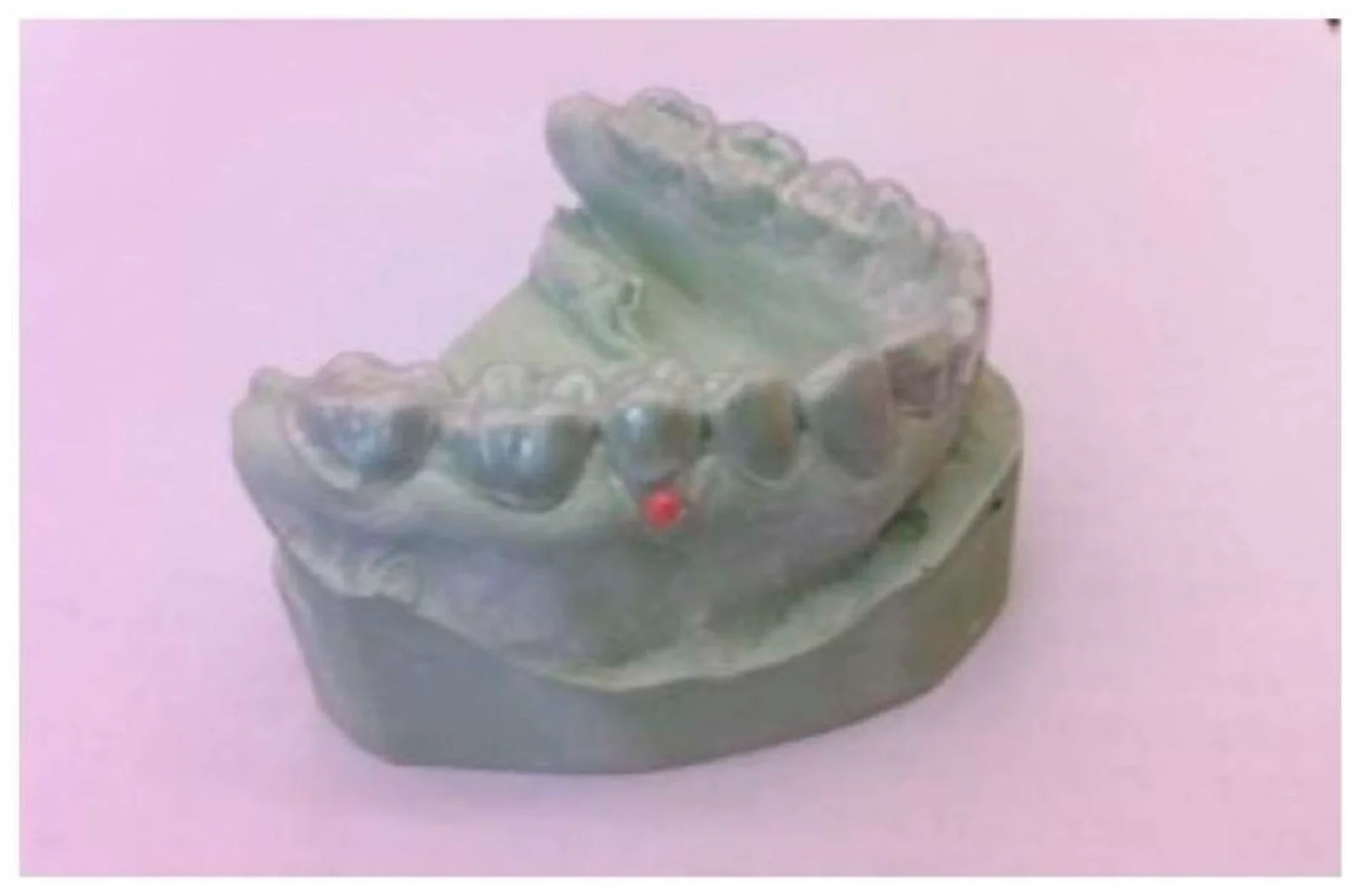
Thermoplastic splint.

A custom thermoplastic splint with an incorporated radiopaque marker (gutta-percha) and a mounting link positioned 3 mm above the marker standardized the measurement location (Figure 2). Splints were fabricated on plaster models from individual alginate impressions, as previously described [24]. The splint was used to mark a buccal gingival site 3 mm apical to the gingival margin. Local anesthesia was administered before measurements to standardize clinical conditions. GT was measured using four methods: CBCT, BS, pulse–echo US, and direct digital CA after flap elevation. In the absence of an independent reference standard, the methods were compared symmetrically. BS and post-elevation CA were included as clinically established direct-contact comparators, although each is subject to method-specific sources of measurement error. Each method was assessed twice by two oral surgeons (A.G. and G.D.).

**Figure 3:**
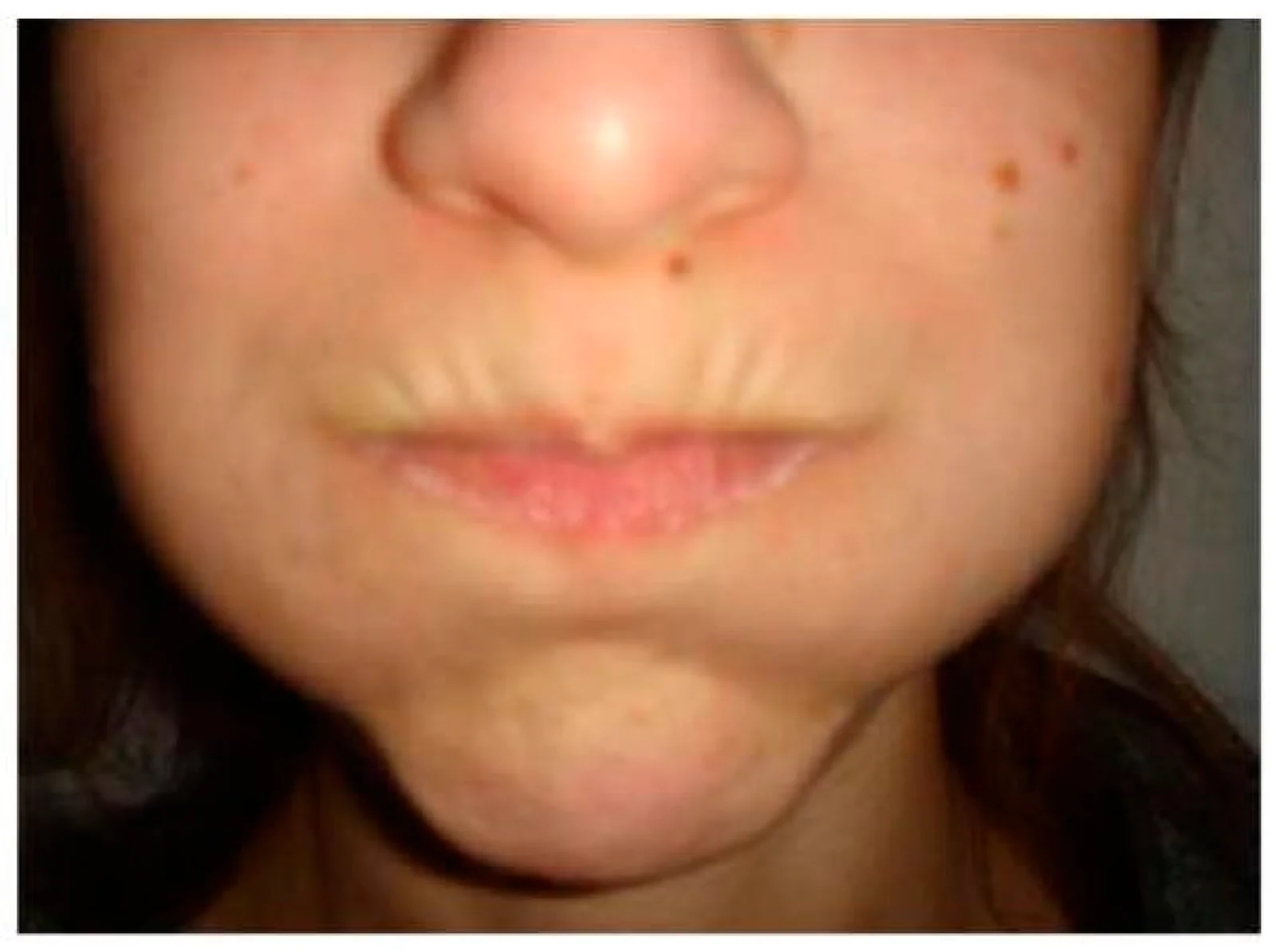
Puffed-cheek maneuver used to separate the facial soft tissues during CBCT acquisition.

CBCT scans (Morita 3D Accuitomo 170, J. MORITA MFG. CORP., Kyoto, Japan) were acquired at 90 kV, 5 mA, an 18 s exposure, a 40 × 40 mm field of view, and a nominal voxel size of 80 micrometers; reconstructed slice thickness was 0.25 mm. The thermoplastic splint remained in situ. Facial soft-tissue separation was achieved using the puffed-cheek method or, when anesthesia prevented the maneuver, manual retraction (Figure 3). These approaches may produce different soft-tissue separation and tension and were therefore considered a potential source of measurement variability. GT was measured at the mounting link using OsiriX software (version 4.0, Pixmeo Sarl, Geneva, Switzerland). Each archived CBCT dataset was evaluated twice by both examiners, with the two reading sessions separated by four weeks; no repeat CBCT acquisition was performed.

**Figure 4:**
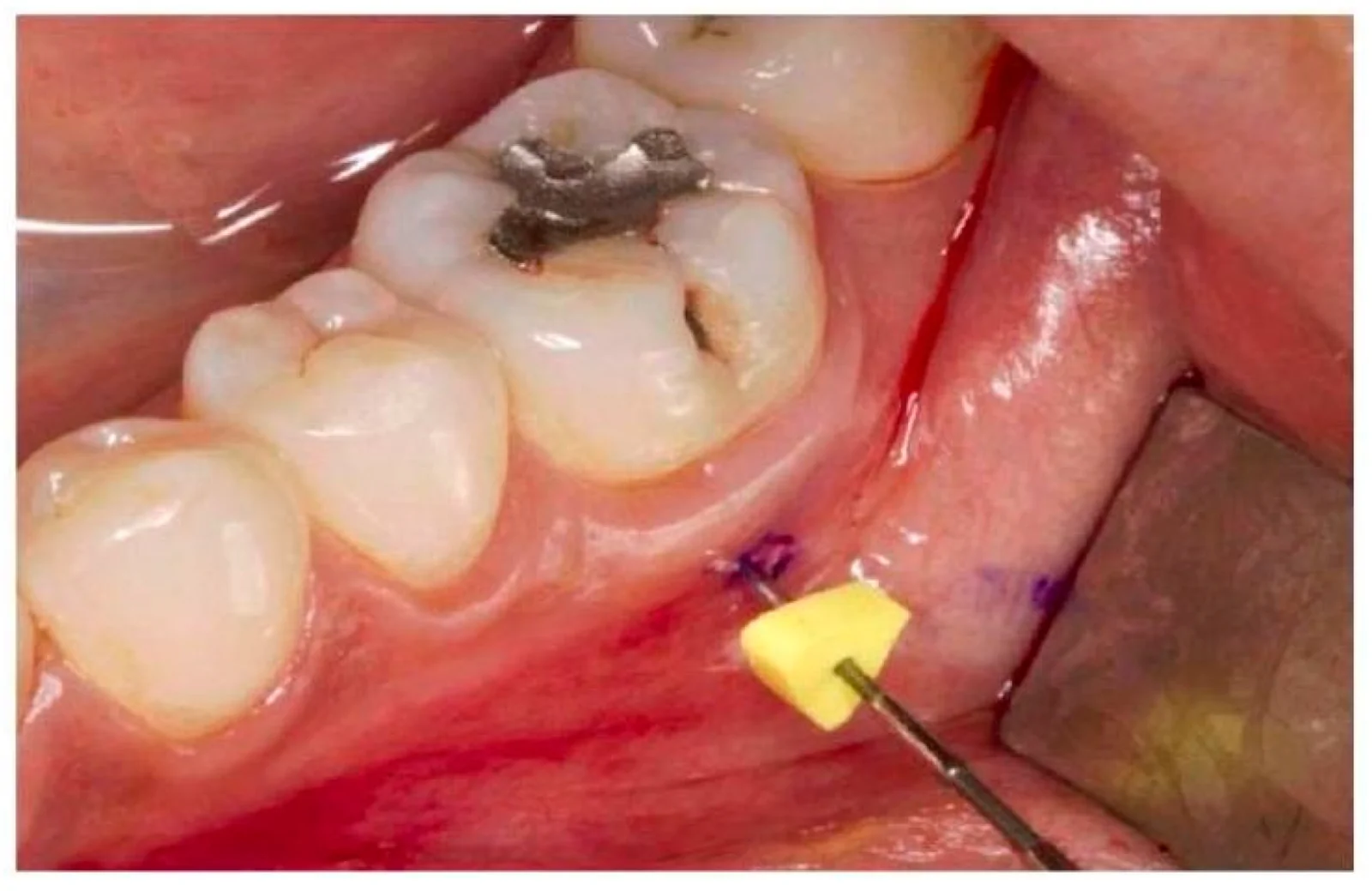
Bone sounding procedure.

**Figure 5:**
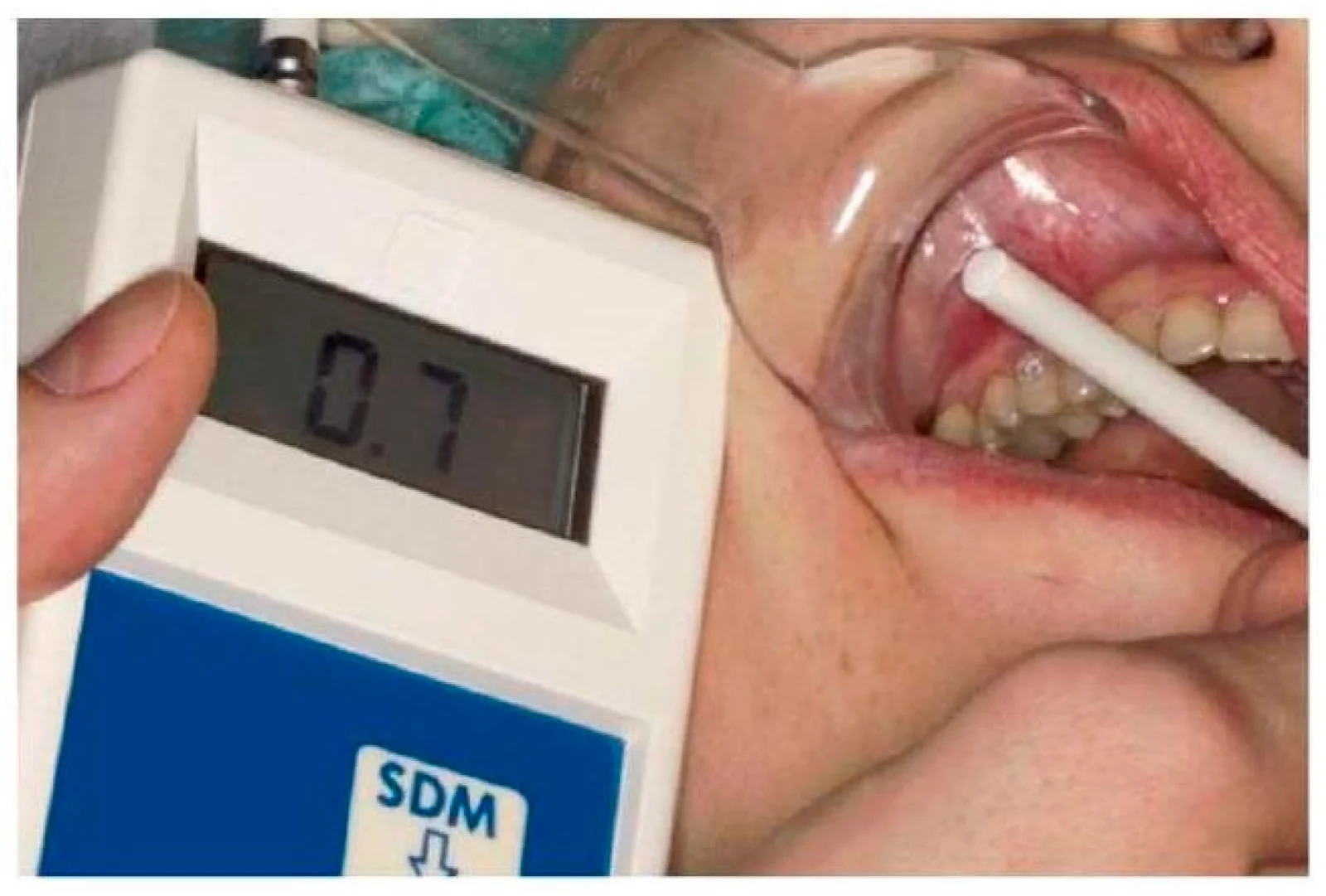
Ultrasonic pulse–echo instrument.

BS was performed immediately after CBCT with the splint in place. An ISO 30 endodontic reamer with a rubber stopper was inserted perpendicular to the gingival surface until bone contact at the marked site (Figure 4), and the distance was read with a digital caliper. For the exploratory phenotype analysis, the threshold used in the archived analysis was applied consistently: GT < 1.1 mm was classified as thin and GT ≥ 1.1 mm as thick. This threshold is close to recently proposed histology-based cutoffs, but phenotype thresholds vary by anatomical landmark and measurement modality [28]. Ultrasound measurements were obtained at the marked site with the Krupp SDM pulse–echo instrument (Austenal Medizintechnik, Cologne, Germany; Figure 5). This earlier-generation A-mode device provides a single-point thickness reading without two-dimensional anatomical visualization and differs substantially from contemporary B-mode, Doppler, quantitative ultrasound, and elastographic systems. During surgery, GT was measured directly after flap elevation with a digital caliper. Each measurement was repeated twice by both examiners.

The examiners were not blinded to the results of the other methods because the four measurements were obtained sequentially at the same marked site during the same clinical session. This design facilitated co-localization but may have introduced assessment bias. The repeated readings used for reliability estimation, including the two CBCT evaluation sessions described in Section 2.2, were performed separately from this single clinical session.

### 2.3. Statistical Analysis

The measurement site was the unit of analysis for the present comparative analysis. The original sample-size planning was based on previous data indicating a mean GT of 1.5 ± 0.4 mm [24], a clinically relevant difference of 0.30 mm, alpha = 0.05, and 80% power. The present analysis comprised 41 evaluable measurement sites. Repeat-level data were used for inter-examiner reliability analyses, and the four observations per method (two examiners × two repetitions) were averaged to generate one site-level estimate for inter-method comparison. Reliability was assessed using a two-way random-effects, absolute-agreement, single-measures intraclass correlation coefficient (ICC(2,1)), in which the four measurements per site (two examiners × two repetitions) were entered as the rater dimension within a single model, together with Cronbach’s alpha. Agreement was summarized using the mean paired difference (comparator minus CBCT), its 95% confidence interval, and Bland–Altman 95% limits of agreement (mean difference ± 1.96 SD), presented both graphically and numerically. A ±0.30 mm clinical comparison margin, based on the clinically relevant difference specified for the original sample-size calculation, was used to interpret the Bland–Altman results. Approximate confidence intervals for the limits of agreement were calculated using the standard error SD × sqrt(3/n). Friedman tests with Dunn-adjusted post hoc comparisons assessed distributional differences, and Spearman rank correlations assessed association; neither analysis was interpreted as evidence of agreement or interchangeability. For exploratory phenotype classification relative to the CBCT category, complete 2 × 2 tables, McNemar exact tests, sensitivity, specificity, positive and negative predictive values, accuracy, and Cohen kappa were calculated. Exact Clopper–Pearson 95% confidence intervals were used for proportions, and asymptotic 95% confidence intervals were used for kappa. Because only four CBCT sites were classified as thin, all phenotype estimates were considered exploratory. Two-sided *p* values < 0.05 were considered statistically significant.

## 3. Results

### 3.1. Study Sample and Reliability

Of the 53 CBCT examinations acquired, 12 (23%) were non-evaluable because the buccal soft tissues could not be reliably delineated, leaving 41 evaluable sites (77%) for analysis. Eleven sites (26.8%) were anterior and 30 (73.2%) posterior; eight (19.5%) were maxillary and 33 (80.5%) mandibular. Inter-examiner reliability, calculated from the repeated measurements, was high for CBCT (ICC 0.89; Cronbach’s alpha 0.90), BS (ICC 0.97; alpha 0.97), pulse–echo US (ICC 0.92; alpha 0.92), and CA (ICC 0.97; alpha 0.97); all *p* values were <0.001. These estimates describe reproducibility among evaluable measurements and do not address the 23% CBCT non-evaluable rate.

### 3.2. Gingival Thickness Measurements

Mean GT was 1.71 ± 0.56 mm with CBCT, 1.52 ± 0.57 mm with BS, 1.55 ± 0.68 mm with pulse–echo US, and 0.99 ± 0.59 mm with CA. Mean paired differences (comparator minus CBCT) were −0.19 ± 0.40 mm for BS, −0.16 ± 0.34 mm for US, and −0.71 ± 0.65 mm for CA. Thus, BS and the tested US device showed smaller average differences from CBCT than CA.

### 3.3. Agreement Analysis

**Figure 6:**
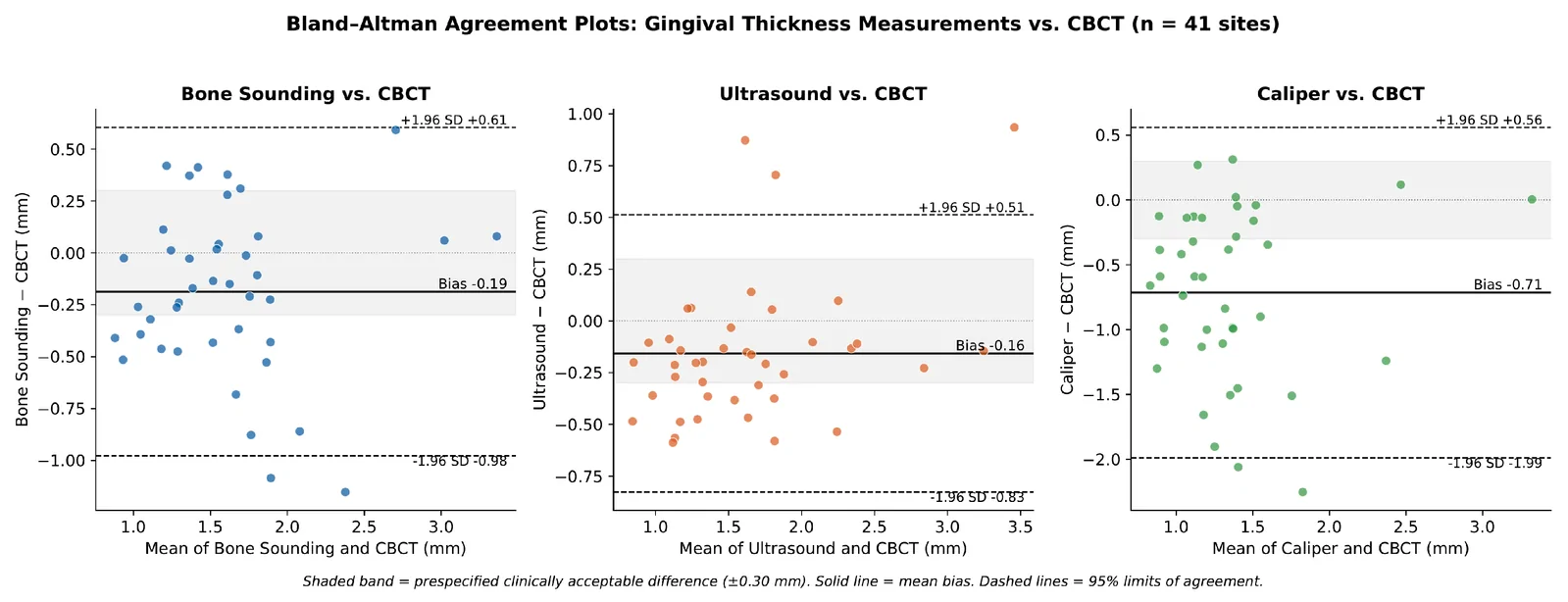
Bland–Altman agreement plots for bone sounding (BS), pulse–echo ultrasound (US), and caliper (CA) measurements versus CBCT (n = 41 sites). Bias is calculated as comparator minus CBCT. Solid line = mean bias; dashed lines = 95% limits of agreement; shaded band = ±0.30 mm clinical comparison margin (see Methods, Section 2.3).

Bland–Altman agreement plots and numerical summaries are presented in Figure 6 and Table 1. The 95% limits of agreement were −0.98 to 0.61 mm for BS, −0.83 to 0.51 mm for pulse–echo US, and −1.99 to 0.56 mm for CA. Although the average biases for BS and US were within the ±0.30 mm comparison margin, the limits of agreement for all three comparisons extended substantially beyond that interval. Therefore, none of the comparator methods met this margin for interchangeability with CBCT.

### 3.4. Secondary Comparative and Correlation Analyses

The Friedman test showed an overall difference among methods (χ^2^ = 54.48; *p* < 0.0001). Dunn-adjusted comparisons found no statistically significant distributional difference between BS and CBCT (*p* = 0.7445), whereas US (*p* = 0.0016) and CA (*p* < 0.0001) differed from CBCT. Spearman correlations were 0.649 for CBCT versus BS, 0.814 for CBCT versus US, and −0.013 for CBCT versus CA. These findings describe distributional differences and association but were not used to infer agreement, equivalence, or interchangeability.

### 3.5. Gingival Phenotype Classification

Using the consistent threshold of <1.1 mm for thin and ≥1.1 mm for thick gingiva, CBCT classified four of 41 sites (9.8%) as thin. BS classified eight sites (19.5%) as thin, pulse–echo US classified 12 (29.3%), and CA classified 29 (70.7%). Complete 2 × 2 counts and exploratory performance estimates relative to the CBCT category are presented in Table 2 and Table 3. BS had 50.0% sensitivity and 83.8% specificity, US had 100% sensitivity and 78.4% specificity, and CA had 75.0% sensitivity and 29.7% specificity. The confidence intervals were wide, particularly for sensitivity, because only four CBCT sites were thin; these estimates must not be interpreted as definitive diagnostic accuracy measures.

## 4. Discussion

This method-comparison study evaluated four techniques at a standardized buccal site. BS and the tested pulse–echo US device had smaller mean differences from CBCT than CA. However, the Bland–Altman limits of agreement for every comparison exceeded the ±0.30 mm comparison margin. Accordingly, a small mean bias or a non-significant rank comparison should not be interpreted as interchangeability. The results support relative comparison of the methods within this cohort, not validation of CBCT or BS as a gold standard.

The ultrasound findings apply specifically to the Krupp SDM single-point pulse–echo device and protocol. Contemporary high-frequency B-mode, Doppler, quantitative, and elastographic systems provide anatomical or functional information not available from this device [29,36,37,38]. The present findings should therefore not be generalized to ultrasonography as a whole. The strong correlation between CBCT and pulse–echo US did not translate into sufficiently narrow limits of agreement, illustrating why correlation alone is inadequate for assessing interchangeability.

CA showed the largest average bias and widest limits of agreement. Tissue compression, manipulation after flap elevation, and variation in operator pressure may contribute to this finding [19,20]. Nevertheless, the present study does not establish that CA is categorically unsuitable; rather, it indicates that this specific protocol produced the least favorable agreement with CBCT among the methods tested.

Several limitations require emphasis. First, this was a single-center study with a modest and anatomically imbalanced sample, and only four sites were classified as thin by CBCT; phenotype performance estimates are therefore exploratory and imprecise. Second, the 23% non-evaluable CBCT rate (12 of 53 scans) was mainly associated with inadequate separation and inward collapse of the buccal soft tissues, particularly in posterior and retromolar regions, resulting in overlapping soft-tissue contours that prevented reliable gingival thickness measurement; this limits the feasibility and generalizability of the evaluated CBCT protocol. Third, the puffed-cheek and manual-retraction techniques may not create equivalent soft-tissue separation. Fourth, no independent reference standard such as histology was available, so the study assesses agreement rather than diagnostic accuracy. Fifth, measurements were sequential, and examiners were not blinded. Sixth, only inter-examiner reliability (ICC, Cronbach’s alpha) is reported; examiner-specific intra-examiner repeatability estimates were not available for this dataset. Seventh, acquisition parameters for the pulse–echo ultrasound device beyond those reported in Section 2.2 (e.g., probe orientation, coupling medium, applied pressure, calibration, and handling of unstable readings) were not documented in the archived protocol. Eighth, data were collected in 2012, and public registration occurred retrospectively in 2025, reflecting the historical nature of the dataset and the later decision to complete and report this comparative analysis; no separate prospectively finalized statistical analysis plan is available beyond the analyses described in the original protocol, so phenotype analyses should be regarded as exploratory. Ninth, the CBCT and pulse–echo ultrasound equipment reflect the technology and clinical protocols available during the 2012 data-collection period and differ from current-generation systems. Nevertheless, the standardized anatomical co-localization of four measurement methods within the same clinical cohort remains methodologically informative. Device-specific performance should therefore not be extrapolated to current-generation systems. Finally, CBCT involves ionizing radiation and should not be undertaken solely for GT measurement without a separate clinical indication; use must follow risk–benefit assessment and the ALARA principle.

Taken together, BS and the tested pulse–echo US device showed smaller average differences from CBCT than CA, but none achieved limits of agreement within the ±0.30 mm comparison margin. The 23% non-evaluable CBCT rate and the device- and protocol-specific nature of the findings should be considered when interpreting their applicability to current clinical systems.

## 5. Conclusions

Within the limitations of this single-center method-comparison study based on clinical data collected in 2012, BS and the tested pulse–echo US device showed smaller average differences from CBCT than direct CA measurement. Nevertheless, all 95% limits of agreement exceeded the ±0.30 mm comparison margin, and no method can be considered interchangeable with CBCT on the basis of these data. Exploratory phenotype estimates were unstable because only four sites were classified as thin. The findings should be interpreted as device- and protocol-specific and, together with the 23% non-evaluable CBCT rate observed in this cohort, do not justify CBCT exposure solely for gingival thickness assessment.

## Figures and Tables

**Table 1:** Numerical Bland–Altman agreement summaries for CBCT versus comparator methods.

Comparator	Mean Bias (mm)	95% CI for Bias	95% Limits of Agreement (mm)	Within ±0.30 mm?
BS	−0.19	−0.32 to −0.06	−0.98 to 0.61 (95% CI: lower −1.19 to −0.77; upper 0.40 to 0.82)	No
Pulse–echo US	−0.16	−0.27 to −0.05	−0.83 to 0.51 (95% CI: lower −1.01 to −0.65; upper 0.33 to 0.69)	No
CA	−0.71	−0.92 to −0.50	−1.99 to 0.56 (95% CI: lower −2.33 to −1.65; upper 0.22 to 0.90)	No

Bias was calculated as comparator minus CBCT. The comparison margin was ±0.30 mm, based on the difference used in the original sample-size calculation. CI, confidence interval; BS, bone sounding; US, ultrasound; CA, caliper.

**Table 2:** Exploratory 2 × 2 phenotype classifications relative to CBCT.

Method	True Thin	False Thick	False Thin	True Thick	Method-Classified Thin
BS	2	2	6	31	8 (19.5%)
Pulse–echo US	4	0	8	29	12 (29.3%)
CA	3	1	26	11	29 (70.7%)

CBCT classified 4 sites as thin and 37 as thick. Thin was defined as <1.1 mm; values ≥ 1.1 mm were classified as thick. CBCT served only as the classification comparator, not an independent gold standard.

**Table 3:** Exploratory phenotype classification performance relative to CBCT, with 95% confidence intervals.

Method	Sensitivity	Specificity	PPV	NPV	Accuracy	Kappa
BS	50.0% (6.8–93.2)	83.8% (68.0–93.8)	25.0% (3.2–65.1)	93.9% (79.8–99.3)	80.5% (65.1–91.2)	0.234 (−0.131 to 0.598)
Pulse–echo US	100% (39.8–100)	78.4% (61.8–90.2)	33.3% (9.9–65.1)	100% (88.1–100)	80.5% (65.1–91.2)	0.414 (0.119 to 0.709)
CA	75.0% (19.4–99.4)	29.7% (15.9–47.0)	10.3% (2.2–27.4)	91.7% (61.5–99.8)	34.1% (20.1–50.6)	0.012 (−0.107 to 0.132)

Values are estimates (95% CI). Exact Clopper–Pearson intervals were used for proportions; asymptotic intervals were used for Cohen kappa. Estimates are unstable because only four CBCT sites were classified as thin. PPV, positive predictive value; NPV, negative predictive value.

## Data Availability

De-identified data supporting the findings of this study may be made available by the corresponding author upon reasonable request, subject to ethics approval and data-protection requirements.
